# A Review on In Vitro Evaluation of Chemical and Physical Digestion for Controlling Gastric Digestion of Food

**DOI:** 10.3390/foods14081435

**Published:** 2025-04-21

**Authors:** Hiroyuki Kozu, Isao Kobayashi, Sosaku Ichikawa

**Affiliations:** 1 Institute of Food Research, NARO, 2-1-12 Kannondai, Tsukuba 305–8642, Ibaraki, Japan; kozu.hiroyuki484@naro.go.jp; 2 Institute of Life and Environmental Sciences, University of Tsukuba, 1-1-1 Tennodai, Tsukuba 305–8572, Ibaraki, Japan

**Keywords:** food digestion control, gastric digestion, gastric peristalsis, in vitro digestion evaluation, chemical digestion, physical digestion, mechanical characteristics

## Abstract

Food digestion in the gastrointestinal is a series of processes consisting of chemical and physical digestion. Recently, developing foods with controlled digestion in the stomach may attract more attention. Hydrogel foods are useful tools for designing foods with controlled digestion because it is relatively easy to design their food characteristics by adjusting the type and content of the additives. This review introduces the latest status of in vitro gastric digestion as a food characterization system. The in vitro evaluation of chemical gastric digestion by gastric acid and digestive enzymes focuses on INFOGEST-standardized gastrointestinal digestion protocols for healthy adults, infants, and older adults. For the in vitro evaluation of physical gastric digestion by peristalsis, the current development of gastrointestinal tract devices that precisely or efficiently simulate the shape of the stomach and gastric peristalsis is described. In addition, we introduce studies that have utilized these devices to investigate the gastric digestion behavior of hydrocolloid foods with different mechanical characteristics.

## 1. Introduction

### 1.1. Food Digestion Process in GI Tract

**Figure 1:**
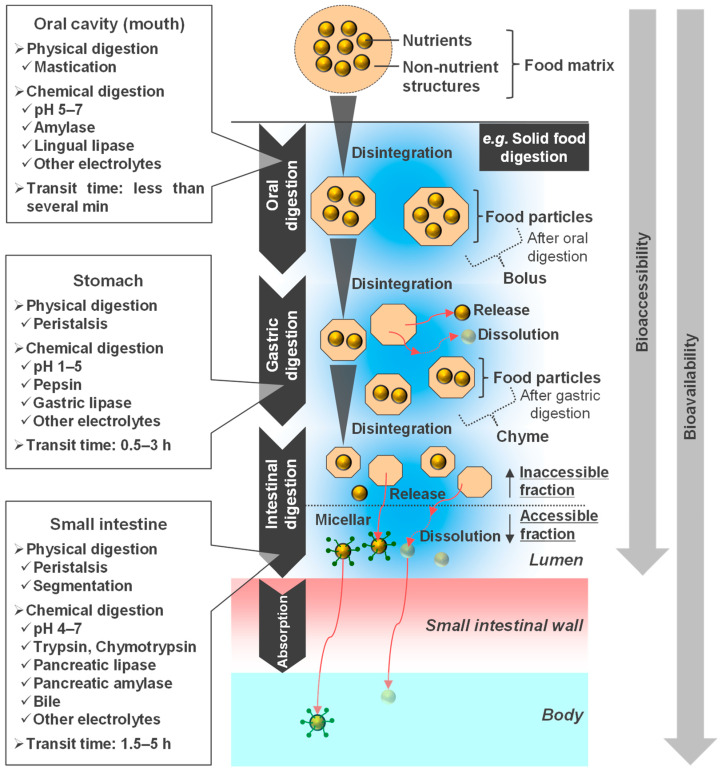
Schematic image of the food digestion process. The ranges of pH and transit times vary slightly in the literature [5,8].

Digestion in the gastrointestinal (GI) tract can be broadly divided into physical and chemical digestion [1]. Physical digestion involves mixing contents and disintegrating food particles, whereas chemical digestion involves the chemical decomposition of food components. In digestion, a food matrix is defined as “nutrient and non-nutrient components of foods and their molecular relations” [2]. A schematic image of the food digestion process is shown in Figure 1. From a food engineering perspective, the digestion process is a collection of unit operations consisting of grinding, mixing, reaction, and separation [3].

Here, the digestive process is outlined with the example of solid foods. Physical oral digestion involves mastication. The force of mastication is on the order of several hundred Newton force, depending on the dental condition [4]. Chemical digestion is the reaction caused by amylase/lingual lipase and saliva [5]. Lingual lipase has also recently been investigated in detail [6]. The pH of saliva is typically 5–7 [5]. The transit time in the oral cavity is less than a few minutes [5]. With mastication, food particles roughly disintegrate to a few millimeters as they mix with saliva and become a bolus [4,7].

**Figure 2:**
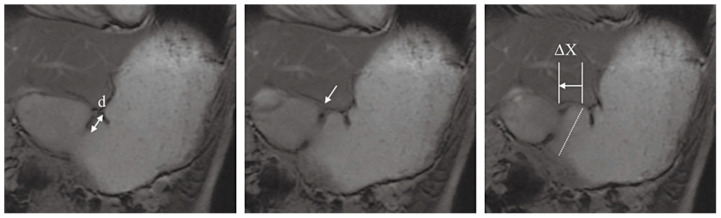
Magnetic resonance imaging of gastric peristalsis; arrows: antral contraction waves; d: the respective deepness of the wave; ΔX: the travel distance of the wave in 20 s; the figure is reproduced from [11] with permission from John Wiley & Sons, Inc.

The stomach is a J-shaped digestive organ with a maximum volume of approximately 4 L in the fed state, consisting mainly of the fundus/body (proximal part) and antrum/pylorus (distal part) [9,10]. A bolus is temporarily stored in the proximal part, transferred to the distal part by gastric wall contraction, and digested in the antrum [10]. Physical digestion in the stomach is triggered by gastric peristalsis in the antrum (Figure 2). Gastric peristalsis is an antral contraction wave that progresses from the proximal part of the stomach to the distal pylorus [11,12]. Peristalsis further disintegrates the food particles and mixes and empties the contents [9,10,13]. The force applied during gastric wall contraction is several Newton force [5,14]. The speed of progression is 1.5–3.0 mm/s, and the frequency is 2.5–3.0 waves/min [1,15,16]. Chemical digestion in the stomach is a reaction caused by pepsin, gastric lipase, and gastric fluid [5]. Pepsin aids in protein hydrolysis by trypsin in the small intestine [17]. Gastric lipase also decomposes a certain amount of lipids in the stomach [18]. The pH of the contents increases from 1 to 2 in the fasting state to approximately 5 after ingestion and then decreases again to the original pH value within a few hours [19,20]. The transit time is generally 0.5–3 h [21,22,23]. The pH and transit times range slightly in the literature [5,8]. Gastric peristalsis empties food particles approximately 2 mm from the pylorus [9,10,24]. Some nutrients in the food particles are released and dissolved [13]. The product of gastric digestion is called chyme [1,9]. The undigested product is emptied by strong stomach wall contractions, known as housekeeper waves [25].

Physical digestion in the small intestine involves the transport of contents by peristalsis and the mixing of contents by segmentation [1,5]. Chemical digestion is the reaction caused by trypsin, chymotrypsin, pancreatic lipase, pancreatic amylase, bile, and intestinal fluid [5]. The pH of the contents is typically 4–7 [26,27]. The transit time is generally 1.5–5 h [22,28]. The pH and transit times range slightly in the literature [5,8]. In small intestinal digestion, nutrients are dissolved from food particles or micellized by bile acids and absorbed by the small intestinal wall, eventually reaching the systemic circulation.

### 1.2. Controlling the Food Digestion Process

In the food digestion process, bioaccessibility is defined as the “proportion of a nutrient that is chemically and physically available for absorption by the small intestine” and bioavailability as the “proportion of a nutrient that is actually absorbed and is available for functionalisation inside the body” (Figure 1) [29]. The definitions of these terms vary in detail in the literature [30,31]. The characteristics of the food matrix can change the amount of internal nutrients released or dissolved into digestive fluids, which can affect bioaccessibility and bioavailability. For example, food matrices with high viscosity or hardness are less likely to be mixed and disintegrated in the GI tract, which may lead to a lower release level and dissolution of internal nutrients. Blood glucose levels vary with the type of food [32]. Even with the same nutrient composition, bread and pasta affect blood glucose levels differently [33]. The blood glucose response varies depending on the processing method of the staple grain [34]. As for rice, the staple food of the Japanese, brown rice also causes a milder rise in blood glucose levels than white rice [35]. Therefore, from a food engineering perspective, “digestion-controlled foods” are expected to behave as intended in the GI tract by adjusting the characteristics of the food matrix. Specifically, it controls the digestion and absorption of target nutrients [36,37,38] and satiety [39,40,41]. The needs of people, including older adults, infants, individuals with obesity, athletes, and patients with gastrointestinal diseases, vary according to age and health status [42,43,44,45]. It is important to design food digestion characteristics that consider each need. In particular, gastric digestion, among GI tracts, is a process that occurs immediately after oral digestion, and the physical and chemical state of food changes drastically.

One example of an application of controlling digestion is food for older adults. In Japan, the percentage of people aged over 65 exceeded 21% of the population in 2007, making it a “super-aging society” [46,47]. In older adults, physiological decline and nutritional deficiencies become chronic, causing age-related chronic diseases such as diabetes, osteoporosis, and dysphagia [48]. The transitional state from health to disease with aging is called frailty, which increases vulnerability owing to a reduced functional reserve [49]. Given this background, the standardization of food products for older adults is progressing, considering the mastication and swallowing abilities of older adults. The International Dysphagia Diet Standardisation Initiative (IDDSI) presented “The IDDSI framework for food textures and drink thickness for people with mastication or swallowing difficulties” in 2019 [48]. However, in the GI tract, the digestive function of the stomach and small intestine is chronically decreased in older adults [50]. Examples are decreased gastric acid secretion/digestive enzyme activity and increased pH of gastric contents [51,52,53,54]. Therefore, food design that considers digestive function in older adults is an area of interest [45].

### 1.3. In Vitro Evaluation of Gastric Digestion

There are three approaches for evaluating gastric digestion: in vivo by human studies, in silico by computer simulation, and in vitro by replacing the digestive environment with laboratory instruments or devices [29]. The advantage of in vivo methods is that actual human data can be obtained, whereas the disadvantages are ethical issues. For this reason, data are currently limited to imaging studies such as magnetic resonance imaging (MRI) [55,56,57]. The application of in silico methods for gastric digestion has the advantage of visualizing mainly flow phenomena inside the stomach [58,59]. However, computer simulations have difficulty analyzing the complex disintegration of solid foods [60].

**Figure 3:**
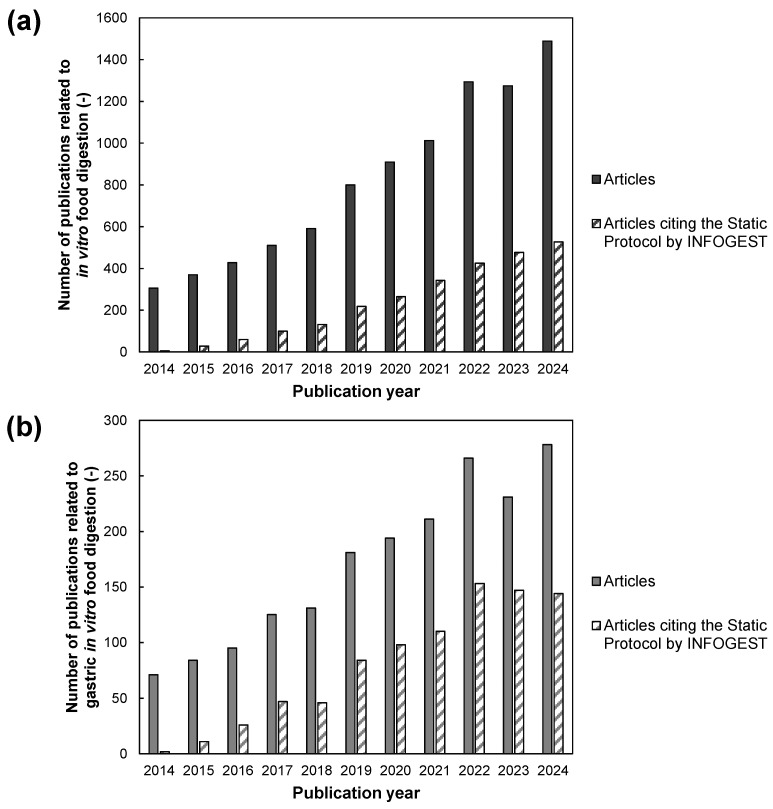
Number of publications related to in vitro food digestion: (**a**) overall scale, (**b**) scale of studies related to gastric digestion. The investigation was conducted on 3 February 2025. The database used was the Web of Science Core Collection. The search queries were as follows: (**a**) ((((PY = (2014–2025)) AND TS = (food AND digest*)) AND TS = (“in vitro”))) AND DT = (Article), (**b**) ((((PY = (2014–2025)) AND TS = (food AND digest*)) AND TS = (“in vitro”)) AND TS = (“stomach” OR “gastric”)) AND DT = (Article). The legend “Articles citing the Static Protocols by INFOGEST” refined the hits from the above queries by limiting them to articles whose cited references include DOIs for either of the following Static Protocols: [66,67].

One useful tool for solving these issues is the in vitro method. This method is free of ethical issues because the digestive environment is replaced by laboratory equipment and devices. In addition, complex phenomena, such as the digestion of solid foods, can be evaluated. Earlier studies on in vitro food digestion began with artificial digestive solutions and glassware [61]. The development of model devices that simulate gastrointestinal motility has been ongoing since 2010 [62]. Currently, research on in vitro digestion is becoming more active, and the number of publications on gastric digestion has increased over the past 20 years (Figure 3). Food design based on in vitro evaluation of gastric and small intestinal digestion has also been discussed [63].

### 1.4. Objectives of This Review

The authors studied the influence of solid food characteristics on gastric digestive behavior by in vitro evaluation [64]. Solid foods derived from natural products are difficult to interpret because of the complex structure of the food matrix. Hydrogel foods are useful as a design tool for digestion-controlled foods, as it is relatively easy to design food characteristics by adjusting the type and content of additives [45]. Hydrogel foods are also useful as a carrier for bioactive compounds [65]. Particular research attention has been paid to macroscopic mechanical characteristics, such as hardness and breaking characteristics. If the degree of disintegration of food in the stomach can be controlled by adjusting the mechanical characteristics of the food, the release and dissolution of nutrients in the food matrix can be altered to control digestion in the stomach and small intestine.

This review presents the latest status of in vitro gastric digestion evaluation systems for foods, dividing them into chemical and physical digestion. First, trends in the standardization of chemical in vitro digestion are described. Next, focusing on physical digestion, recent developments in digestion devices that simulate gastric peristalsis and studies of gastric digestion of hydrogel foods using such devices are introduced.

## 2. Standardization of In Vitro Chemical Digestion Protocols

### 2.1. INFOGEST Static In Vitro Digestion Protocol

A trend in chemical gastric digestion in vitro is standardization. Before 2014, each laboratory independently determined the chemical conditions for in vitro digestion (e.g., pH and enzyme activity) using medical data [61,68]. A standardized static in vitro digestion protocol was released in 2014 by INFOGEST (a consortium of food digestion researchers) [67]. Table 1 summarizes the protocols proposed by INFOGEST and other participating researchers, including the variations described below.

**Table 1:** In vitro digestion protocols proposed by INFOGEST.

INFOGEST Protocol	Proposed Paper	Subject	Static/Semi-dynamic * ^3^	Static Protocol : Major Items Standardized Other Protocols : Major Differences from the Static Protocol	Recent Application Examples [Ref.] * ^6^
Static Protocol	[66,67] * ^2^	Healthy adults	Static	Oral, gastric, and intestinal phase Simulated digestive fluids * ^4^ Mixing ratio of simulated digestive fluids to test sample * ^5^ Duration in each phase	Vitamin D-loaded emulsions [69] Cricket-derived protein [70] Whey protein gels [71]
Semi-dynamic Protocol	[72]	Healthy adults	Semi-dynamic	Examples of apparatus required for the dynamic protocol Secretion flow rate of simulated gastric fluid and emptying flow rate of gastric contents during the gastric phase	Cooked lentils [73] Cheddar cheese [74] Soybean protein [75]
Static Infant Protocol * ^1^	[76]	Full-term newborns (28 days old)	Static	Oral phase omitted Higher ratio of test sample to simulated digestive fluids, different target pH of the mixtures Lower activity of digestive enzymes and bile salt concentration	Soy and pea proteins [77] Skim milk [78] Human milk and infant formula [79]
Static Older Adult Protocol	[80]	Adults over 65 years old	Static	Mastication protocol Higher target pH of simulated gastric juice and test sample mixture Longer duration in gastric phase Lower activity of some digestive enzymes and bile salt concentration	Lentil grains and quinoa seed flours [81] Cream cheese [82] Protein derived from different sources [83]

* ^1^ Proposed by INFOGEST participants. * ^2^ The first standard protocol presented in 2014 was validated for inter-laboratory differences in 2016 and subsequently published in Nature Protocols in 2019 [66,67,84]. * ^3^ Static: no secretion of simulated digestive fluids/emptying of contents. Semi-dynamic: continuous secretion of simulated gastric fluids and emptying of gastric contents. * ^4^ Electrolyte concentration, enzyme activity, and pH in the preparation of artificial saliva, gastric fluid, and intestinal fluid were proposed in the literature. * ^5^ Target pH of the mixture was specified. * ^6^ Listed studies applying all or part of the protocol. Examples were selected as much as possible based on the following criteria: balance of main nutrients, colloid-related test samples, and most recent studies.

The first release was the static digestion protocol for healthy adults (hereafter referred to as the “Static Protocol”). The protocol is based on medical data from healthy adults and standardizes the composition of a reasonable artificial digestive fluid, the mixing ratio of sample to artificial digestive fluid, and digestion duration for the oral, stomach, and small intestine phases. It involves batch digestion experiments for the oral, gastric, and small intestine phases. The first standard protocol presented in 2014 was validated for inter-laboratory differences in 2016 and subsequently published in Nature Protocols in 2019 [66,67,84]. The number of articles related to in vitro digestion citing the Static Protocol has gradually increased since 2014, when the first protocol was published, and has exceeded several hundred in recent years (Figure 3). Note that the numbers in this figure are simply statistical data on the number of articles that cited the Static Protocols by INFOGEST.

In vitro digestion tests are now prominently based on the Static Protocol, with over 2000 original papers in the Web of Science database citing INFOGEST 2.0 (as of 3 February 2025). A very recent example is the application to digestion studies of processed foods such as Vitamin D-loaded emulsions, whey protein gels, and cricket-derived proteins [69,70,71]. The Static Protocol has also been applied to probiotics, toxicity testing, and 3D-printed food [85,86,87,88]. Other applications of the Static Protocol to digestion studies involving various foods have been reviewed [89,90]. Furthermore, the Static Protocol is frequently compared to the INFOGEST protocol variant described below [73,81,82,83,91,92,93,94,95,96,97,98,99,100,101,102,103]. The primary benefit of standardizing protocols is that experimental results can be compared between different laboratories. In fact, reports of inter-laboratory in vitro digestion studies of the same skim milk powder indicate that more harmonized inter-laboratory protein hydrolysis electrophoresis results are obtained with the INFOGEST Static Protocol than with the in-house protocol [84]. This standardization makes benchmarking easier and allows experimental data to be used even more effectively.

### 2.2. Variation of INFOGEST Protocol

Variations in the INFOGEST protocol are shown after the second line of Table 1. First, a protocol that changed part of the Static Protocol into a semi-dynamic process was proposed in 2020 (hereafter referred to as the “Semi-dynamic Protocol”) [72]. Based on the Static Protocol, this protocol changes the gastric phase from batch to continuous. The protocol proposes the conditions for continuous artificial gastric fluid secretion, gastric content emptying, and examples of the apparatus needed for continuous digestion experiments. For example, in vitro digestion of cooked lentils, cheddar cheese, and soybean protein has been reported using the Semi-dynamic Protocol [73,74,75]. One advantage of this protocol is that it provides continuous artificial gastric fluid, which prevents an increase in the pH of the gastric contents derived from the digested sample [104,105]. This protocol also allows for time-series fraction-by-fraction analysis of gastric contents [74,104,106]. A report comparing in vitro digestion of cooked lentils with the Static and Semi-dynamic Protocols indicated that the Semi-dynamic Protocol reduced the rate of protein digestion [73]. In the Static Protocol, the pH of the sample and artificial gastric fluid mixture is adjusted to 3 [67]. On the other hand, the pH is temporarily higher than 3 in the Semi-dynamic Protocol because the artificial gastric fluid is supplied to the sample gradually [73,104,105]. This results in a slow digestion rate in the early stages of digestion due to reduced activity of pepsin in the artificial gastric fluid and insufficient denaturation of proteins [73]. This environment is considered a more appropriate representation of actual gastric digestion, especially when evaluating the chemical digestion of proteins.

In vitro digestion studies in infants have been a research focus for over a decade [107,108]. In 2018, a protocol was proposed by part of the INFOGEST research group that modified the Static Protocol to the digestive environment of infants (hereafter referred to as the “Static Infant Protocol”) [76]. The Static Infant Protocol is based on medical data from full-term newborns (28 days old) and has the following major changes compared to the Static Protocol: omission of the oral phase to account for the liquid nature of the infant formula; higher ratio of sample to artificial digestive fluid; higher target pH of the mixture of artificial digestive fluid and sample in the gastric phase and slightly lower in the small intestine phase; and lower digestive enzyme activity and bile acid concentration in the gastric and small intestine phases. In vitro digestion of skim milk, human milk, infant formula, and soy/pea proteins has been reported using the Static Infant Protocol [77,78,79]. The focus was on digestion experiments on samples related to milk products. The Static Infant Protocol has reduced pepsin activity in the gastric phase and lipase activity in the intestinal phase by about a tenth compared to the Static Protocol; the pH of the gastric phase is also higher at 5.3 [76]. Since milk products, as the main food of infants, have a protein-emulsified structure of fat globules, this modification in pepsin and lipase activity alters the in vitro digestion. When comparing both protocols in the infant formula, the level of protein digestion in the Static Infant Protocol is less than half, and the level of lipid digestion is also reduced; in another study using the Infant formula, the bioaccessibility of DHA in samples after small intestinal digestion was decreased; differences have also been reported in the release of iron ions in fat globules [76,95,96]. Because the differences between these protocols are not negligible, the Static Infant Protocol is considered essential when evaluating the chemical digestion of foods for infants.

In vitro digestion in older adults has been studied for over a decade [109,110]. In 2023, a protocol was proposed that modified the Static Protocol to the digestive environment of older adults (hereafter referred to as the “Static Older Adult Protocol”) [80]. The Static Older Adult Protocol is based on medical data from adults over 65 years old and has the following major changes compared to the Static Protocol: the mastication protocol has changed in the oral phase, the target pH of the mixture of artificial gastric digestive fluid and sample in the gastric phase and the digestion duration has increased, and the digestive enzyme activity and bile acid concentration in the gastric and small intestine phases has decreased. In vitro digestion of lentil grains/quinoa seeds flours, cream cheese, and protein derived from different sources has been reported using the Static Older Adult Protocol [81,82,83]. The degree of protein or lipid digestion in these samples is reduced to up to half of the Static Protocol [81,82,83]. Since the Static Infant Protocol suppresses protein digestion by about a tenth in some cases, the reduction level of the Static Older Adult Protocol seems to be mild [76]. This may be because the level of setting change in the Static Older Adult Protocol is mild compared to the Static Infant Protocol: enzyme activity is about half, and the pH of the gastric phase is slightly higher at 3.7 [76,80]. However, the Static Older Adult Protocol could be recommended for the evaluation of the digestion of foods intended for older adults since the studies reported significant differences between the two protocols [81,82,83].

In addition, individual research groups have reported various arrangements of the INFOGEST protocol [90]. For example, in vitro digestion of exocrine pancreatic insufficiency is based on the Static Protocol [111]. More recently, the following INFOGEST protocol arrangements have been developed: in vitro digestion of infants of different ages based on the Static Infant Protocol [78]; in vitro digestion in older patients with mastication abnormalities based on the Static Older Adult Protocol [102]; and application of the Semi-dynamic Protocol to the Static Infant Protocol [78,112,113] and to the Static Older Adult Protocol [73]. In vitro digestion simulating sex differences has also been proposed [114].

As described above, in vitro chemical digestion studies are becoming easier to compare among research groups owing to the standardization of protocols. The influence of the chemical characteristics of foods (molecular, interfacial, etc.) on chemical digestion can now be modeled and evaluated appropriately. Recently, protocols have been modified to suit different age groups and health conditions. Standardized evaluation protocols are expected to allow for more accurate benchmarking among laboratories. It should be noted that these protocols are suitable for evaluating the chemical digestion of food components. The protocols presented are supposed to use ground food samples; the food sample and the artificial digestive fluid are mixed almost homogeneously in the vessel during the test. [66,67], while in actual gastric digestion, solid foods are gradually disintegrated and mixed with gastric fluid by peristalsis [9,10,13]. Such physical gastric digestion could be particularly affected by the mechanical characteristics of solid foods. Thus, these protocols may not always be effective as a means of evaluating the impact of the physical characteristics of solid foods on gastric digestion. The next section describes an in vitro evaluation focused on gastric physical digestion.

## 3. Recent Studies on In Vitro Evaluation of Physical Digestion in the Stomach

### 3.1. Current GI Tract Devices Simulating Gastric Peristalsis

Compared to chemical digestion, the evaluation of the physical digestion of foods, especially solid foods, has not been standardized in vitro. This is because the gastrointestinal motility responsible for physical digestion in the stomach and small intestine cannot be simulated using ordinary laboratory equipment. Therefore, a device that simulates gastrointestinal motility (hereafter referred to as the “GI tract device”) is used to evaluate physical food digestion. The TNO Gastrointestinal Model (TIM) and the Dynamic Gastric Model (DGM) are representative early GI tract devices that simulate the segmental motion of the stomach using the water pressure drive and the up-and-down motion of a piston, respectively [115,116,117]. The Human Gastric Simulator (HGS) and Gastric Digestion Simulator (GDS) simulate the progressing waves of gastric peristalsis by compressing the flexible wall with rollers and moving the rollers from top to bottom [118,119]. Various GI tract devices are currently being developed by various research groups [14,120].

**Figure 4:**
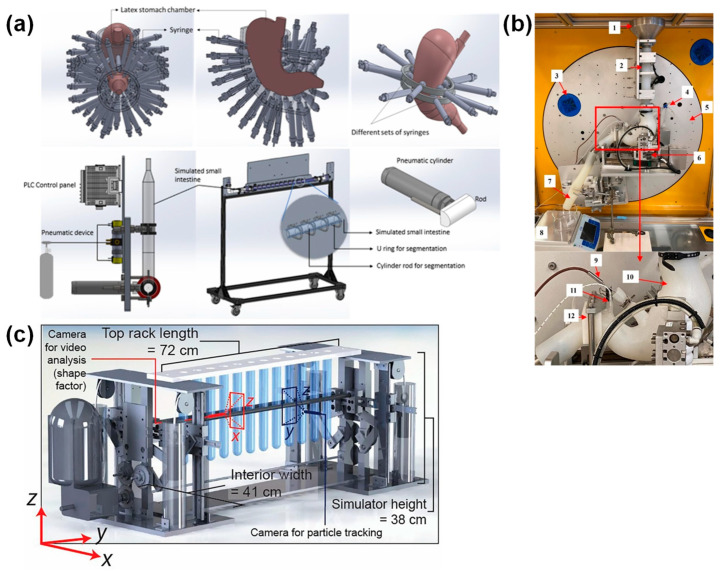
Examples of recent GI tract devices that simulate gastric peristalsis: (**a**) Gastric Simulation Model (GSM) at the top and Small Intestinal Simulator (SIS) at the bottom [92]; (**b**) NEar-Real Digestive Tract (NERDT) (commercialized name of Dynamic In Vitro Human Stomach System [DHS-IV]) (see the original article for the detailed names of the areas labeled with numbers) [123]; (**c**) multi-module peristaltic simulator [124]. All figures are reproduced from the literature with permission from Elsevier B.V.

A trend limited to gastric GI tract devices is the enhancement of peristalsis simulation. A recent example of a gastric GI tract device is shown in Figure 4. The gastric wall contracts in all directions via peristalsis in the human stomach. However, when peristalsis is simulated in a device by partially compressing a flexible wall with moving parts (e.g., rollers), a so-called “dead space” is not compressed. The Rope-driven in vitro Human Stomach (RD-IV-HSM), the Human Gastric Simulator v2.0 (HGS v2.0), and the Gastric Simulation Model (GSM) reduce dead space by mechanically compressing the J-shaped flexible chamber of the stomach from nearly all directions using ropes, clamps, and cylinders, respectively (e.g., see GSM in Figure 4a) [92,121,122].

The tilt angle of the stomach is an important parameter. The Dynamic In Vitro Human Stomach System (DHS-IV; currently commercialized under the name NEar-Real Digestive Tract (NERDT)) was intended to simulate the accurate emptying of gastric contents by driving peristalsis. This device has a mechanism that can control the tilt angle of the J-shaped gastric chamber at any given time (Figure 4b) [123,125]. Differences in the emptying rate of contents by peristalsis have been observed when the tilt angle was varied stepwise during digestion experiments [125].

GI tract devices using a single gastric vessel are time-consuming and require a high throughput. A recently developed multi-module peristaltic simulator can generate peristalsis-like movements in multiple tubes simultaneously by arranging multiple cylindrical, flexible tubes and compressing them with a roller at once (Figure 4c) [124]. Although this is a basic study analyzing the flow of liquid content, it is expected to be a high-throughput digestion experiment in principle.

Other GI tract devices that simulate the J-shape of the stomach and the contraction waves of peristalsis include the Artificial Gastric Digestive System (AGDS), Artificial Stomach Response Kit (ARK^®^), In vitro Mechanical Gastric System (IMGS), Realistic Gastric Model (RGM), and Soft Robotic Gastric Simulator (SoGut) [126,127,128,129,130]. Furthermore, the composition of artificial digestive fluids described in the INFOGEST protocol in the previous section is often applied in digestion experiments using a GI tract device: AGDS, GDS, GSM, HGS, NERDT (DHS-IV), and RGM [91,92,123,126,127,131]. For example, a study using HGS compared the results of gastric digestion of cooked couscous under four conditions: the INFOGEST Static/Semi-dynamic Protocol, the United States Pharmacopeia (USP) protocol, and the UC Davis standard protocol [91]. The USP protocol with lower amounts of pepsin tended to have a lower disintegration level of the couscous particles. It should be mentioned that there was no superiority or inferiority between protocols. As described above, GI tract devices that simulate gastric peristalsis are advancing in hardware and software.

### 3.2. Utilization of Agar Gel Beads for Force Validation in GI Tract Devices

The GI tract device, which simulates gastric peristalsis with the mechanical driving force of a motor, destroys food regardless of the degree of hardness if the force exerted by the roller driven by the motor is too strong. Therefore, it is essential to adjust the force exerted on solid foods by gastric peristalsis simulated by the GI tract device for in vitro evaluation of the physical gastric digestion of solid foods [14]. Force validation using agar gel beads is a promising approach. In a well-known human study, 12.7 mm agar gel beads of different agar concentrations or fracture forces were swallowed by subjects without mastication, and the number of beads that disintegrated in the gastric peristalsis was counted from MRI images [132]. According to this study, the half-life of agar gel bead disintegration increased when the fracture force was between 0.65–0.78 N. Table 2 summarizes the studies that validated the force of the gastric GI tract device using agar gel beads with different agar concentrations or fracture forces, similar to this human study.

The DGM, which simulates peristalsis by the up-and-down motion of the piston, was able to reproduce the increasing disintegration half-life of agar gel beads within the same range of fracture force as in the human study [116]. The RD-IV-HSM, which is a pioneering device that precisely simulates the j-shape of the stomach, with wrinkles on the inner wall and rope-driven peristalsis progressing waves, was unable to disintegrate agar gel beads at all fracture forces from 0.53–0.90 N [122]. This indicates that even if the geometry of the device is similar to that of the human stomach, it may not always be able to reproduce the forces required for the disintegration of solid food owing to various factors such as construction and operating mechanisms on the device. GDS and AGDS with a roller-driven peristalsis mechanism increased the half-life of agar gel bead disintegration at the boundaries of agar concentration or fracture force corresponding to the human study [127,133]. GDS and AGDS fine-tune the force required for the disintegration of solid food by changing the softness of the rollers and the distance between the rollers and the stomach vessel, respectively.

The GI tract device is a machine, and it is difficult to simulate all functions of the human stomach. Although the DGM does not simulate the progressing waves of gastric peristalsis, it is useful regarding the disintegration evaluation of solid foods. Although RD-IV-HSM could not simulate the disintegration of agar gel beads by peristalsis, its successor, the near real Dynamic In Vitro Human Stomach (new DIVHS), is expected to evaluate gastric emptying behavior accurately [122,134]. It is important to understand the intentions behind the evaluation of each device.

### 3.3. Possibility of Using Hydrogel Food and Gastric GI Tract Device to Study Gastric Digestion Control

Hydrogel foods effectively control gastric digestion because their mechanical characteristics can be easily artificially adjusted using gelling agents and processes [45]. Gastric peristalsis physically fractures the gel [132]. Therefore, a GI tract device that simulates gastric peristalsis is necessary for appropriate in vitro evaluation. In this section, we introduce a study investigating the gastric digestion behavior of hydrogel foods using a GI tract device that simulates gastric peristalsis and discuss the possibility of controlling gastric digestion.

Table 3 summarizes the examples of studies that used HGS, GDS, IMGS, and AGDS to simulate gastric peristalsis. In previous HGS studies, WPI emulsion gels were prepared by gelling soybean oil emulsions with whey protein isolate (WPI) [135,136,137]. Changing the internal droplet size or the NaCl concentration added during WPI gelation changes the mechanical characteristics of the gel (hardness, fracture force/strain, etc.) [135,138]. They used a grinder to simulate the mastication of the oral phase. Interestingly, the so-called “harder” gels with higher mechanical characteristics had smaller gel particle sizes after the mastication process. However, the harder gel resulted in a slower degree of disintegration and protein hydrolysis after the digestion experiments and a different rate of fatty acid release after conventional in vitro small intestinal digestion experiments.

Our research group, using GDS, focused on the effect of gel fracture stress/strain on physical gastric digestion. Non-protein hydrogel foods mixed with agar and native gellan gum were prepared [133,139]. Gels with constant fracture stress and different strains, and vice versa, were prepared by adjusting the mixing ratio and concentration of these gelling agents. GDS digestion experiments were performed on these hydrogel foods cut into 5 mm cubes as a simulated mastication process. The results showed that gels with a fracture strain above a certain value hardly disintegrated during gastric digestion, regardless of fracture stress. In the region of small fracture strain, the smaller the fracture stress, the greater the degree of gel disintegration [133]. The percentage of emptied starch decreased with disintegration in cornstarch-containing hydrogels with high fracture stress/strain [139].

The IMGS study used a sample of sunflower oil emulsion gelled with WPI [140]. In this study, the mechanical characteristics of WPI emulsion gels were varied by changing the pH during gel preparation and the pressure during emulsification. They used WPI gels masticated by human volunteers for gastric digestion experiments. The harder the gel, the longer the mastication time was set. The degree of protein hydrolysis in the gels after the IMGS gastric digestion experiment varied according to the mechanical characteristics of the gels. The degree of fatty acid release after the conventional in vitro small intestinal digestion experiment was slower in harder gels. In vitro gastric digestion in a stirred beaker performed on similar samples showed smaller differences in the mechanical characteristics of the WPI gels compared to the IMGS results. This indicates that simulating peristalsis is important to evaluate the effects of the mechanical properties of the gels on gastric digestion.

AGDS was used to study *tofu*, a gel-like food made from soy curd [141]. The mechanical characteristics of *tofu* differ depending on the type of coagulant (glucono-d-lactone (GLD) or CaSO_4_) used in its preparation. The degree of disintegration of *tofu* particles after oral/gastric digestion and the viscosity of digesta varied depending on the mechanical characteristics of *tofu*. The degree of protein hydrolysis of the gastric emptying fraction was slower in harder *tofu.*

Gel fracture force/stress and strain have been measured by several research groups [133,135,136,137,139,140]. Fracture strain, which represents the amount of compressive deformation required for the gel to fracture, may be the point at which disintegration occurs when gel particles are compressed by gastric peristalsis [133]. In other studies, gels with higher fracture strains had a relatively low degree of gastric digestion [135,140]. However, this may be most apparent when the gels used in gastric digestion experiments are relatively large, approximately 5 mm cube in size [133]. If the gel is pre-crushed, the conditions are already different in the initial state of the gastric digestion experiment because the particle size after the simulated mastication process changes depending on the mechanical characteristics [136,137]. Although other characteristics, such as hardness and Young’s modulus, also need to be considered, these values are difficult to benchmark because their definitions and units change depending on the measurement [136,137,140,141]. Protein gels, such as WPI and *tofu*, are also chemically digested by pepsin in the stomach. In addition to the macroscopic mechanical characteristics, the penetration of pepsin due to differences in microscopic microstructures can also affect digestion.

The major limitations of GI tract devices and hydrogels to simulate gastric digestion include the phenomenon of physical and chemical gastric digestion without fracture. For example, studies using GDS to evaluate gastric digestion of rice and bread have shown that particle swelling occurs [142,143]. The study using NERDT has shown that intragastric coagulation of milk products occurs [123]. These cannot be predicted by macroscopic fracture characteristics and require consideration of the chemical composition and microstructure of the food. Furthermore, foods that are extremely soft or have very small particle sizes after the mastication process may be better considered as fluids. In the HGS digestion experiments of heat-set milk protein gels, the gel selected as the soft gel was semi-solid, and its rheological characteristics and complex modulus were measured [144]. Temporal changes in the mechanical characteristics of foods immersed in artificial gastric fluid may be another factor that controls gastric digestion [145]. It should be noted that hydrogel digestion represents only a portion of the complex gastric digestion phenomenon. Further research is needed to clarify the relationship between the mechanical characteristics of hydrogel-based foods and gastric digestion. At least, we know that the degree of gastric digestion of hydrogel foods with adjusted mechanical characteristics changes when they are subjected to digestion experiments using a GI tract device that simulates gastric peristalsis. The use of these GI tract devices will clarify the influence of the mechanical characteristics of food on the physical digestion of the stomach.

## 4. Summary, Prospects, and Conclusions

This review presents the latest status of in vitro gastric digestion evaluation systems for foods. For the in vitro evaluation of chemical gastric digestion by gastric acid and digestive enzymes, standardization of gastrointestinal digestion protocols, primarily INFOGEST, is in progress. The first standardized digestion protocols for healthy adults evolved to consider digestion in infants and older adults. As these studies develop, the chemical digestion of food can be controlled. The in vitro evaluation of physical gastric digestion, such as the disintegration of solid foods and mixing of contents by gastric peristalsis, has not yet been standardized. The development of GI tract devices that accurately or efficiently simulate gastric geometry and peristalsis is ongoing. Some studies utilizing these devices, limited to hydrocolloid foods, have revealed the potential for controlling gastric digestion by adjusting the mechanical characteristics of the food.

The standardization of in vitro evaluation of chemical digestion has preceded physical digestion; however, some common protocols may be necessary for physical digestion. If physical digestion can be standardized, it will be possible to evaluate physical digestion as well as chemical digestion, suitable for infants and older adults. In order to reduce costs and increase versatility, it may be necessary to consider ways in which the phenomenon of disintegration and mixing by gastric peristalsis can be replaced reasonably with instruments that can be easily available in the laboratory. Furthermore, the integration of standardized chemical and physical digestion methods and guidelines that stipulate their use may be in the future.

Both food characteristics and digestion processes are extremely complex. Food products in aging and super-aged societies are increasingly standardized, considering mastication and swallowing functions. As for next-generation foods, the development of digestion-controlled foods may attract attention in the future. Low-cost in vitro digestion evaluation is extremely effective in efficiently evaluating the gastric digestion of diverse foods. It will be necessary to foster a common understanding among researchers over time and move closer to a systematic understanding of the factors determining digestive behavior.

## Figures and Tables

**Table 2:** Case study of compression force validation in gastric GI tract devices using agar gel beads.

Gastric GI Tract Device	Methods for Simulating Peristalsis	Agar Gel Beads Used for Validation * ^1^			Ref.
		Diameter (mm)	Agar Concentration (wt%)	Fracture Strength (N)	
Cf. Human stomach	Progressing wave: antral contraction wave	12.7	0.75–3	0.15–0.90	[132]
Dynamic Gastric Model (DGM)	Up-and-down stoke of piston	12.7	1.51, 1.89, 2.39, 3.0	0.53, 0.65, 0.78, 0.90	[116]
Rope-driven in vitro Human Stomach (RD-IV-HSM)	Progressing wave: rope-driven system	12	-	0.15–0.65	[122]
Gastric Digestion Simulator (GDS)	Progressing wave: roller-driven system	12.7	1.50, 1.89, 2.39, 3.00	-	[133]
Artificial Gastric Digestive System (AGDS)	Progressing wave: roller-driven system	12.7	2.0, 2.5, 3.0, 3.5, 4.0	0.35, 0.50, 0.65, 0.80, 0.95	[127]

* ^1^ Ranges of values and significant figures in the table are quoted verbatim from the original papers.

**Table 3:** Case study of the gastric digestion behavior of hydrogel foods with different mechanical characteristics using a GI tract device that simulates gastric peristalsis.

Hydrogel Food to Be Digested				Gastric GI tract Device * ^6^	Major Experimental Results	Ref.
Name (Major Materials)	Mechanical Characteristics		Oral Phase * ^1^			
	Factors That Vary the Characteristics	Characteristics Reported * ^1,2^				
Whey protein isolated (WPI) emulsion gel (soybean oil emulsion; whey protein isolated)	Oil droplet size in WPI gel	Storage modulus (kPa): (A) 63.7; (B) 71.4; (C) 84.0 Fracture force (N): (A) 4.0; (B) 7.5; (C) 12.0 Fracture strain (%): (A) 31.3; (B) 46.2; (C) 50.6	Crushed using a grinder Mixed with artificial saliva Median size: < 1 mm	Human Gastric Simulator (HGS)	Emulsion gels with smaller droplet diameters exhibited harder mechanical characteristics Slow disintegration and protein hydrolysis in the case of harder gel	[135]
WPI emulsion gel (soybean oil emulsion; whey protein isolated)	Concentration of NaCl to be added during preparation	Hardness (N)*3: (A) 19.2; (B) 69.9 Young’s modulus (kPa) * ^3^: (A) 19.7; (B) 228.8 Recoverable energy (%) * ^3^: (A) 47.7; (B) 30.8 Fracture force (N) * ^3^: (A) 3.9; (B) 17.2 Fracture strain (%) * ^3^: (A) 63.1; (B) 68.1 Toughness (J/m^2^) * ^3^: (A) 102.9; (B) 327.6	Crushed using a grinder Mixed with artificial saliva Median size (mm): (A) 3.84; (B) 0.97	HGS	Emulsion gels with higher NaCl concentration exhibited harder mechanical characteristics Harder gels had smaller particle sizes after the oral phase Slow disintegration rate in the case of harder gel Fatty acid release in conventional in vitro intestine digestion of the emptied fraction differed between the soft and hard gels	[136,137]
Hydrogel (agar; native-type gellan gum)	Mixing ratio of agar and native-type gellan gum Concentration of each gelling agent	Fracture stress (kPa): (A) 21.1; (B) 23.4; (C) 22.6; (D) 39.6; (E) 40.0; (F) 37.5; (G) 54.1; (H) 56.7; (I) 62.7; (J) 76.1 Fracture strain (%): (A) 26.5; (B) 37.3; (C) 60.8; (D) 28.9; (E) 38.1; (F) 63.9; (G) 29.7; (H) 41.2; (I) 68.2; (J) 30.6	Manually cut into 5-mm cubes Mixed with artificial saliva	Gastric Digestion Simulator (GDS)	The overall trend was that the higher the total concentration of the two gelling agents, the higher the gel fracture stress, and the higher the ratio of native-type gellan gum, the higher the gel fracture strain rate Gels with a fracture strain above a certain value rarely fractured during gastric digestion In the region of low fracture strain, the lower the fracture stress, the higher the degree of fracture	[133]
Hydrogel containing starch (agar; native-type gellan gum; corn starch)	Mixing ratio of agar and native-type gellan gum Concentration of each gelling agent	Fracture stress (kPa): (A) 17.4; (B) 57.6; (C) 20.9; (D) 55.5 Fracture strain (%): (A) 25.4; (B) 28.5; (C) 53.7; (D) 57.4	Manually cut into 5-mm cubes Mixed with artificial saliva	GDS	Mechanical characteristics and disintegration behavior of the gels showed the same trends as in the above study Starch gels with high fracture stress and/or strain decreased the percentage of starch emptied	[139]
WPI emulsion gel (sunflower oil emulsion; whey protein isolated)	pH during preparation Pressure during emulsification	Hardness (N): (A) 10.81; (B) 8.97; (C) 11.31; (D) 13.27 Cohesiveness (-): (A) 0.44; (B) 0.46; (C) 0.89; (D) 0.86 Chewiness (N): (A) 4.57; (B) 3.77; (C) 8.52; (D) 7.40 Stress at break (kPa) * ^4^: (A) 21.70; (B) 23.43; (C) 76.21; (D) 94.96 Strain at break (-) * ^5^: (A) 0.21; (B) 0.24; (C) 0.75; (D) 0.76	Mastication by a human volunteer (emulsion gels prepared at higher pH set longer mastication time) Mixed with artificial saliva	In vitro Mechanical Gastric System (IMGS)	Emulsion gels prepared at higher pH had higher levels of mechanical characteristics In the small intestinal digestion experiment of emptied fractions, > the degree of protein hydrolysis depended on the mechanical characteristics of the emulsion gel prepared> harder emulsion gels had lower release rates of free fatty acids	[140]
*Tofu* (gel-like food made of soy curd)	Different coagulant: glucono-δ-lactone (GLD) or CaSO_4_	Hardness (g): (A) 23.1; (B) 105.2 Resilience (%): (A) 3.6; (B) 8.0 Chewiness (g): (A) 1.2; (B) 7.9	Crushed using a stirrer Mixed with artificial saliva	Artificial Gastric Digestive System (AGDS)	*Tofu* prepared with CaSO_4_ exhibited harder mechanical characteristics than *tofu* prepared with GLD *Tofu* with different mechanical characteristics had different particle size distributions after oral/gastric digestion and viscosity of digeta The degree of protein hydrolysis of the gastric emptying fraction was lower in the harder *tofu*	[141]

* ^1^ Symbols (A, B, C, etc.) indicate identical samples within each reference. * ^2^ Names, units, and decimal places are quoted directly from the original papers (standard deviation is omitted; the same term may have different units and definitions in the different literature). * ^3^ Reported in their previous study [138]. * ^4^ Synonymous with “Fracture stress”. * ^5^ Synonymous with “Fracture strain”. * ^6^ GI tract devices were limited to those that simulate the progressive wave of the gastric peristalsis, and minor changes in the device were omitted.

## Data Availability

The original contributions presented in the study are included in the article; further inquiries can be directed to the corresponding author.

## References

[1] Bornhorst G.M., Singh R.P. (2014). Gastric Digestion in Vivo and in Vitro: How the Structural Aspects of Food Influence the Digestion Process. Annu. Rev. Food Sci. Technol..

[2] Unger A.L., Astrup A., Feeney E.L., Holscher H.D., Gerstein D.E., Torres-Gonzalez M., Brown K. (2023). Harnessing the Magic of the Dairy Matrix for Next-Level Health Solutions: A Summary of a Symposium Presented at Nutrition 2022. Curr. Dev. Nutr..

[3] Bornhorst G.M., Gouseti O., Wickham M.S.J., Bakalis S. (2016). Engineering Digestion: Multiscale Processes of Food Digestion. J. Food Sci..

[4] Cichero J.A.Y. (2020). Evaluating Chewing Function: Expanding the Dysphagia Field Using Food Oral Processing and the IDDSI Framework. J. Texture Stud..

[5] Sensoy I. (2021). A Review on the Food Digestion in the Digestive Tract and the Used in Vitro Models. Curr. Res. Food Sci..

[6] Abodi M., Mazzocchi A., Risé P., Marangoni F., Agostoni C., Milani G.P. (2025). Salivary Fatty Acids in Humans: A Comprehensive Literature Review. Clin. Chem. Lab. Med..

[7] Jalabert-Malbos M.-L., Mishellany-Dutour A., Woda A., Peyron M.-A. (2007). Particle Size Distribution in the Food Bolus after Mastication of Natural Foods. Food Qual. Prefer..

[8] Nandhra G.K., Chaichanavichkij P., Birch M., Scott S.M. (2023). Gastrointestinal Transit Times in Health as Determined Using Ingestible Capsule Systems: A Systematic Review. J. Clin. Med..

[9] Kong F., Singh R.P. (2008). Disintegration of Solid Foods in Human Stomach. J. Food Sci..

[10] Liu W., Jin Y., Wilde P.J., Hou Y., Wang Y., Han J. (2021). Mechanisms, Physiology, and Recent Research Progress of Gastric Emptying. Crit. Rev. Food Sci. Nutr..

[11] Baba S., Sasaki A., Nakajima J., Obuchi T., Koeda K., Wakabayashi G. (2009). Assessment of Gastric Motor Function by Cine Magnetic Resonance Imaging. J. Gastroenterol. Hepatol..

[12] Gopirajah R., Raichurkar K.P., Wadhwa R., Anandharamakrishnan C. (2016). The Glycemic Response to Fibre Rich Foods and Their Relationship with Gastric Emptying and Motor Functions: An MRI Study. Food Funct..

[13] Somaratne G., Ferrua M.J., Ye A., Nau F., Floury J., Dupont D., Singh J. (2020). Food Material Properties as Determining Factors in Nutrient Release during Human Gastric Digestion: A Review. Crit. Rev. Food Sci. Nutr..

[14] Li Y., Kong F. (2022). Simulating Human Gastrointestinal Motility in Dynamic in Vitro Models. Compr. Rev. Food Sci. Food Saf..

[15] Kwiatek M.A., Steingoetter A., Pal A., Menne D., Brasseur J.G., Hebbard G.S., Boesiger P., Thumshirn M., Fried M., Schwizer W. (2006). Quantification of Distal Antral Contractile Motility in Healthy Human Stomach with Magnetic Resonance Imaging. J. Magn. Reson. Imaging.

[16] O’Grady G., Gharibans A.A., Du P., Huizinga J.D. (2021). The Gastric Conduction System in Health and Disease: A Translational Review. Am. J. Physiol. -Gastrointest. Liver Physiol..

[17] Rivera del Rio A., Keppler J.K., Boom R.M., Janssen A.E.M. (2021). Protein Acidification and Hydrolysis by Pepsin Ensure Efficient Trypsin-Catalyzed Hydrolysis. Food Funct..

[18] Iddir M., Porras Yaruro J.F., Larondelle Y., Bohn T. (2021). Gastric Lipase Can Significantly Increase Lipolysis and Carotenoid Bioaccessibility from Plant Food Matrices in the Harmonized INFOGEST Static in Vitro Digestion Model. Food Funct..

[19] Gardner J.D., Ciociola A.A., Robinson M. (2002). Measurement of Meal-Stimulated Gastric Acid Secretion by in Vivo Gastric Autotitration. J. Appl. Physiol..

[20] Malagelada J.-R., Longstreth G.F., Summerskill W.H.J., Go V.L.W. (1976). Measurement of Gastric Functions During Digestion of Ordinary Solid Meals in Man. Gastroenterology.

[21] Camilleri M., Malagelada J.R., Brown M.L., Becker G., Zinsmeister A.R. (1985). Relation between Antral Motility and Gastric Emptying of Solids and Liquids in Humans. Am. J. Physiol..

[22] Graff J., Brinch K., Madsen J.L. (2000). Simplified Scintigraphic Methods for Measuring Gastrointestinal Transit Times. Clin. Physiol..

[23] Lin H.C., Prather C., Fisher R.S., Meyer J.H., Summers R.W., Pimentel M., Mccallum R.W., Akkermans L.M.A., Loening-Baucke V., Transit A.T.F.C. (2005). on G. Measurement of Gastrointestinal Transit. Dig. Dis. Sci..

[24] Kelly K.A. (1980). Gastric Emptying of Liquids and Solids: Roles of Proximal and Distal Stomach. Am. J. Physiol..

[25] Coupe A.J., Davis S.S., Evans D.F., Wilding I.R. (1991). Correlation of the Gastric Emptying of Nondisintegrating Tablets with Gastrointestinal Motility. Pharm. Res..

[26] Evans D.F., Pye G., Bramley R., Clark A.G., Dyson T.J., Hardcastle J.D. (1988). Measurement of Gastrointestinal PH Profiles in Normal Ambulant Human Subjects. Gut.

[27] Kalantzi L., Goumas K., Kalioras V., Abrahamsson B., Dressman J.B., Reppas C. (2006). Characterization of the Human Upper Gastrointestinal Contents Under Conditions Simulating Bioavailability/Bioequivalence Studies. Pharm. Res..

[28] Brinch K., Larsson H.B., Madsen J.L. (1999). A Deconvolution Technique for Processing Small Intestinal Transit Data. Eur. J. Nucl. Med..

[29] Le Feunteun S., Verkempinck S., Floury J., Janssen A., Kondjoyan A., Marze S., Mirade P.-S., Pluschke A., Sicard J., van Aken G. (2021). Mathematical Modelling of Food Hydrolysis during in Vitro Digestion: From Single Nutrient to Complex Foods in Static and Dynamic Conditions. Trends Food Sci. Technol..

[30] Dima C., Assadpour E., Dima S., Jafari S.M. (2020). Bioavailability and Bioaccessibility of Food Bioactive Compounds; Overview and Assessment by in Vitro Methods. Compr. Rev. Food Sci. Food Saf..

[31] Carbonell-Capella J.M., Buniowska M., Barba F.J., Esteve M.J., Frígola A. (2014). Analytical Methods for Determining Bioavailability and Bioaccessibility of Bioactive Compounds from Fruits and Vegetables: A Review. Compr. Rev. Food Sci. Food Saf..

[32] Bornet F.R., Billaux M.S., Messing B. (1997). Glycaemic Index Concept and Metabolic Diseases. Int. J. Biol. Macromol..

[33] Granfeldt Y., Bjorck I., Hagander B. (1991). On the Importance of Processing Conditions, Product Thickness and Egg Addition for the Glycaemic and Hormonal Responses to Pasta: A Comparison with Bread Made from “Pasta Ingredients”. Eur. J. Clin. Nutr..

[34] Musa-Veloso K., Poon T., Harkness L.S., O’Shea M., Chu Y. (2018). The Effects of Whole-Grain Compared with Refined Wheat, Rice, and Rye on the Postprandial Blood Glucose Response: A Systematic Review and Meta-Analysis of Randomized Controlled Trials. Am. J. Clin. Nutr..

[35] Cheng Z., Qiao D., Zhao S., Zhang B., Lin Q., Xie F. (2022). Whole Grain Rice: Updated Understanding of Starch Digestibility and the Regulation of Glucose and Lipid Metabolism. Compr. Rev. Food Sci. Food Saf..

[36] Norton J.E., Gonzalez Espinosa Y., Watson R.L., Spyropoulos F., Norton I.T. (2015). Functional Food Microstructures for Macronutrient Release and Delivery. Food Funct..

[37] Bao C., Jiang P., Chai J., Jiang Y., Li D., Bao W., Liu B., Liu B., Norde W., Li Y. (2019). The Delivery of Sensitive Food Bioactive Ingredients: Absorption Mechanisms, Influencing Factors, Encapsulation Techniques and Evaluation Models. Food Res. Int..

[38] Qazi H.J., Ye A., Acevedo-Fani A., Singh H. (2024). Delivery of Encapsulated Bioactive Compounds within Food Matrices to the Digestive Tract: Recent Trends and Future Perspectives. Crit. Rev. Food Sci. Nutr..

[39] Ho I.H., Matia-Merino L., Huffman L.M. (2015). Use of Viscous Fibres in Beverages for Appetite Control: A Review of Studies. Int. J. Food Sci. Nutr..

[40] Kristensen M., Jensen M.G. (2011). Dietary Fibres in the Regulation of Appetite and Food Intake. Importance of Viscosity. Appetite.

[41] Jin Y., Wilde P.J., Hou Y., Wang Y., Han J., Liu W. (2023). An Evolving View on Food Viscosity Regulating Gastric Emptying. Crit. Rev. Food Sci. Nutr..

[42] Norton J.E., Wallis G.A., Spyropoulos F., Lillford P.J., Norton I.T. (2014). Designing Food Structures for Nutrition and Health Benefits. Annu. Rev. Food Sci. Technol..

[43] Bourlieu C., Menard O., Bouzerzour K., Mandalari G., Macierzanka A., Mackie A.R., Dupont D. (2014). Specificity of Infant Digestive Conditions: Some Clues for Developing Relevant in Vitro Models. Crit. Rev. Food Sci. Nutr..

[44] Oustamanolakis P., Tack J. (2012). Dyspepsia: Organic versus Functional. J. Clin. Gastroenterol..

[45] Calligaris S., Moretton M., Melchior S., Mosca A.C., Pellegrini N., Anese M. (2022). Designing Food for the Elderly: The Critical Impact of Food Structure. Food Funct..

[46] Kido Y. (2015). The Issue of Nutrition in an Aging Society. J. Nutr. Sci. Vitaminol..

[47] Muramatsu N., Akiyama H. (2011). Japan: Super-Aging Society Preparing for the Future. Gerontologist.

[48] Qin Y., Pillidge C., Harrison B., Adhikari B. (2024). Pathways in Formulating Foods for the Elderly. Food Res. Int..

[49] Sugie M., Harada K., Nara M., Kugimiya Y., Takahashi T., Kitagou M., Kim H., Kyo S., Ito H. (2022). Prevalence, Overlap, and Interrelationships of Physical, Cognitive, Psychological, and Social Frailty among Community-Dwelling Older People in Japan. Arch. Gerontol. Geriatr..

[50] Lovat L.B. (1996). Age Related Changes in Gut Physiology and Nutritional Status. Gut.

[51] Baron J.H. (1963). Studies of Basal and Peak Acid Output with an Augmented Histamine Test. Gut.

[52] Vellas B., Balas D., Moreau J., Bouisson M., Senegas-Balas F., Guidet M., Ribet A. (1988). Exocrine Pancreatic Secretion in the Elderly. Int. J. Pancreatol..

[53] Feldman M., Cryer B., McArthur K.E., Huet B.A., Lee E. (1996). Effects of Aging and Gastritis on Gastric Acid and Pepsin Secretion in Humans: A Prospective Study. Gastroenterology.

[54] Russell T.L., Berardi R.R., Barnett J.L., Dermentzoglou L.C., Jarvenpaa K.M., Schmaltz S.P., Dressman J.B. (1993). Upper Gastrointestinal PH in Seventy-Nine Healthy, Elderly, North American Men and Women. Pharm. Res..

[55] de Jonge C.S., Smout A.J.P.M., Nederveen A.J., Stoker J. (2018). Evaluation of Gastrointestinal Motility with MRI: Advances, Challenges and Opportunities. Neurogastroenterol. Motil..

[56] Lu K.-H., Liu Z., Jaffey D., Wo J.M., Mosier K.M., Cao J., Wang X., Powley T.L. (2022). Automatic Assessment of Human Gastric Motility and Emptying from Dynamic 3D Magnetic Resonance Imaging. Neurogastroenterol. Motil..

[57] Wang X., Alkaabi F., Choi M., Di Natale M.R., Scheven U.M., Noll D.C., Furness J.B., Liu Z. (2024). Surface Mapping of Gastric Motor Functions Using MRI: A Comparative Study between Humans and Rats. Am. J. Physiol. -Gastrointest. Liver Physiol..

[58] Ishida S., Miyagawa T., O’Grady G., Cheng L.K., Imai Y. (2019). Quantification of Gastric Emptying Caused by Impaired Coordination of Pyloric Closure with Antral Contraction: A Simulation Study. J. R. Soc. Interface.

[59] Kuhar S., Lee J.H., Seo J.-H., Pasricha P.J., Mittal R. (2022). Effect of Stomach Motility on Food Hydrolysis and Gastric Emptying: Insight from Computational Models. Phys. Fluids.

[60] Palmada N., Hosseini S., Avci R., Cater J.E., Suresh V., Cheng L.K. (2023). A Systematic Review of Computational Fluid Dynamics Models in the Stomach and Small Intestine. Appl. Sci..

[61] McClements D.J., Li Y. (2010). Review of in Vitro Digestion Models for Rapid Screening of Emulsion-Based Systems. Food Funct..

[62] Guerra A., Etienne-Mesmin L., Livrelli V., Denis S., Blanquet-Diot S., Alric M. (2012). Relevance and Challenges in Modeling Human Gastric and Small Intestinal Digestion. Trends Biotechnol..

[63] Duijsens D., Pälchen K., Guevara-Zambrano J.M., Verkempinck S.H.E., Infantes-Garcia M.R., Hendrickx M.E., Van Loey A.M., Grauwet T. (2022). Strategic Choices for in Vitro Food Digestion Methodologies Enabling Food Digestion Design. Trends Food Sci. Technol..

[64] Kobayashi I., Kozu H., Wang Z., Isoda H., Ichikawa S. (2017). Development and Fundamental Characteristics of a Human Gastric Digestion Simulator for Analysis of Food Disintegration. Jpn. Agric. Res. Q. JARQ.

[65] Li M., He X., Zhao R., Shi Q., Nian Y., Hu B. (2022). Hydrogels as Promising Carriers for the Delivery of Food Bioactive Ingredients. Front. Nutr..

[66] Brodkorb A., Egger L., Alminger M., Alvito P., Assunção R., Ballance S., Bohn T., Bourlieu-Lacanal C., Boutrou R., Carrière F. (2019). INFOGEST Static in Vitro Simulation of Gastrointestinal Food Digestion. Nat. Protoc..

[67] Minekus M., Alminger M., Alvito P., Ballance S., Bohn T., Bourlieu C., Carrière F., Boutrou R., Corredig M., Dupont D. (2014). A Standardised Static in Vitro Digestion Method Suitable for Food-an International Consensus. Food Funct..

[68] Hur S.J., Lim B.O., Decker E.A., McClements D.J. (2011). In Vitro Human Digestion Models for Food Applications. Food Chem..

[69] Abik F., Ho T.M., Lehtonen M., Philo M., Booth C., Mandalari G., Wilde P.J., Mikkonen K.S. (2024). The Two-Faced Functionality of Birch Glucuronoxylan in an Emulsion-Based Carrier of Vitamin D3. Food Hydrocoll..

[70] de Matos F.M., Rasera G.B., de Castro R.J.S. (2024). Multifunctional Properties of Peptides Derived from Black Cricket (Gryllus Assimilis) and Effects of in Vitro Digestion Simulation on Their Bioactivities. Food Res. Int..

[71] Liu D., Janssen A.E.M., Smeets P.A.M., Stieger M. (2025). Impact of Microstructure of Whey Protein Gels on in Vitro Gastric Protein Digestion Is Sustained after Oral Structural Breakdown by Mastication. Food Hydrocoll..

[72] Mulet-Cabero A.-I., Egger L., Portmann R., Ménard O., Marze S., Minekus M., Le Feunteun S., Sarkar A., Grundy M.M.-L., Carrière F. (2020). A Standardised Semi-Dynamic in Vitro Digestion Method Suitable for Food—An International Consensus. Food Funct..

[73] Duijsens D., Verkempinck S.H.E., Somers E., Hendrickx M.E.G., Grauwet T. (2024). From Static to Semi-Dynamic in Vitro Digestion Conditions Relevant for the Older Population: Starch and Protein Digestion of Cooked Lentils. Food Funct..

[74] Daniloski D., Page R.M., Lamichhane P., Fitzpatrick C.J., Vasiljevic T., Brodkorb A., Timlin M., Murphy J.P., O’Callaghan T.F., McCarthy N.A. (2024). Cheddar Cheese Production, Structure and in-Vitro Semi-Dynamic Gastric Digestion: The Role of β-Casein Phenotype. Food Res. Int..

[75] Han C., Xu Z., Wu K., Wang J., Guo J., Yang X. (2025). Study on Gastric Digestion Behavior of Phytase-Treated Soybean Protein: A Semi-Dynamic Digestion Method. Food Chem..

[76] Ménard O., Bourlieu C., De Oliveira S.C., Dellarosa N., Laghi L., Carrière F., Capozzi F., Dupont D., Deglaire A. (2018). A First Step towards a Consensus Static in Vitro Model for Simulating Full-Term Infant Digestion. Food Chem..

[77] Tang J., Wichers H.J., Hettinga K.A. (2023). Glycation of Soy and Pea Proteins Influences Infant Gastric Digestibility More than Intestinal Digestibility. Food Hydrocoll..

[78] Miltenburg J., Bastiaan-Net S., Hoppenbrouwers T., Wichers H., Hettinga K. (2024). Gastric Clot Formation and Digestion of Milk Proteins in Static in Vitro Infant Gastric Digestion Models Representing Different Ages. Food Chem..

[79] Komatsu Y., Wada Y., Shibasaki T., Kitamura Y., Ehara T., Nakamura H., Miyaji K. (2024). Comparison of Protein Digestibility of Human Milk and Infant Formula Using the INFOGEST Method under Infant Digestion Conditions. Br. J. Nutr..

[80] Ménard O., Lesmes U., Shani-Levi C.S., Araiza Calahorra A., Lavoisier A., Morzel M., Rieder A., Feron G., Nebbia S., Mashiah L. (2023). Static in Vitro Digestion Model Adapted to the General Older Adult Population: An INFOGEST International Consensus. Food Funct..

[81] Sánchez-García J., Muñoz-Pina S., García-Hernández J., Tárrega A., Heredia A., Andrés A. (2024). Protein Digestibility and ACE Inhibitory Activity of Fermented Flours in Older Adults and Standard Gastrointestinal Simulation. Food Res. Int..

[82] Lavoisier A., Chevalier S., Henry G., Ossemond J., Harel-Oger M., Garric G., Dupont D., Morzel M. (2024). Impact of Age on the Digestion of Cream Cheese Formulated with Opposite Caseins to Whey Proteins Ratios: An in Vitro Study. Food Res. Int..

[83] Qiu Z., Shi Y., Zheng Y., Shi W., Zhang L., Yin M., Wang X. (2025). Comparison of in Vitro Digestive Characteristics of Proteins from Different Sources in Simulated Elderly Gastrointestinal Conditions. Food Chem..

[84] Egger L., Ménard O., Delgado-Andrade C., Alvito P., Assunção R., Balance S., Barberá R., Brodkorb A., Cattenoz T., Clemente A. (2016). The Harmonized INFOGEST in Vitro Digestion Method: From Knowledge to Action. Food Res. Int..

[85] Quiroz-Eraso S., Paola Rodriguez-Castaño G., Quintanilla-Carvajal M.X., Acosta-González A. (2024). Microencapsulation of Fat-Removing Lactobacillales and Polyphenols from *Theobroma cacao* L. as a Combined Strategy for Intestinal Removal of Free Fatty Acids Evaluated by Simulated in Vitro Digestion. J. Funct. Foods.

[86] Chen Y., Wang R., Xu M. (2024). Metabolomics Analysis for Unveiling the Toxicological Mechanism of Silver Nanoparticles Using an In Vitro Gastrointestinal Digestion Model. ACS Nanosci. Au.

[87] Sartori A.G.d.O., Saliba A.S.M.C., Bitencourt B.S., Guedes J.S., Torres L.C.R., de Alencar S.M., Augusto P.E.D. (2023). Anthocyanin Bioaccessibility and Anti-Inflammatory Activity of a Grape-Based 3D Printed Food for Dysphagia. Innov. Food Sci. Emerg. Technol..

[88] Zhou Q., Nan X., Zhang S., Zhang L., Chen J., Li J., Wang H., Ruan Z. (2023). Effect of 3D Food Printing Processing on Polyphenol System of Loaded Aronia Melanocarpa and Post-Processing Evaluation of 3D Printing Products. Foods.

[89] Zhou H., Tan Y., McClements D.J. (2023). Applications of the INFOGEST In Vitro Digestion Model to Foods: A Review. Annu. Rev. Food Sci. Technol..

[90] Colombo R., Ferron L., Frosi I., Papetti A. (2021). Advances in Static in Vitro Digestion Models after the COST Action Infogest Consensus Protocol. Food Funct..

[91] Subramanian P., Nadia J., Paul Singh R., Bornhorst G.M. (2023). Comparison of Four Digestion Protocols on the Physical Characteristics of Gastric Digesta from Cooked Couscous Using the Human Gastric Simulator. Food Funct..

[92] Li Y., Xu R., Xiu H., Feng J., Jin Park H., Prabhakar H., Kong F. (2022). Effect of Cinnamon on Starch Hydrolysis of Rice Pudding: Comparing Static and Dynamic in Vitro Digestion Models. Food Res. Int..

[93] Lavoisier A., Morzel M., Chevalier S., Henry G., Jardin J., Harel-Oger M., Garric G., Dupont D. (2023). In Vitro Digestion of Two Protein-Rich Dairy Products in the Ageing Gastrointestinal Tract. Food Funct..

[94] Wang K., Liu D., Tao X., Zhang J., Huppertz T., Regenstein J.M., Liu X., Zhou P. (2023). Decalcification Strongly Affects in Vitro Gastrointestinal Digestion of Bovine Casein Micelles under Infant, Adult and Elderly Conditions. Food Hydrocoll..

[95] Barbosa B.S.T., Garcia-Rojas E.E. (2022). Double Emulsions as Delivery Systems for Iron: Stability Kinetics and Improved Bioaccessibility in Infants and Adults. Curr. Res. Food Sci..

[96] Marques M.C., Perina N.P., Mosquera E.M.B., Tomé T.M., Lazarini T., Mariutti L.R.B. (2021). DHA Bioaccessibility in Infant Formulas and Preschool Children Milks. Food Res. Int..

[97] Torcello-Gómez A., Dupont D., Jardin J., Briard-Bion V., Deglaire A., Risse K., Mechoulan E., Mackie A. (2020). Human Gastrointestinal Conditions Affect in Vitro Digestibility of Peanut and Bread Proteins. Food Funct..

[98] Torcello-Gómez A., Dupont D., Jardin J., Briard-Bion V., Deglaire A., Risse K., Mechoulan E., Mackie A. (2020). The Pattern of Peptides Released from Dairy and Egg Proteins Is Highly Dependent on the Simulated Digestion Scenario. Food Funct..

[99] Chen X., Fan R., Wang X., Zhang L., Wang C., Hou Z., Li C., Liu L., He J. (2024). In Vitro Digestion and Functional Properties of Bovine β-Casein: A Comparison between Adults and Infants. Food Res. Int..

[100] Wang Z., Liu D., Hong X., Tao X., Zhang J., Zhang J., Hou Y., Wu T., Liu X., Zhou P. (2024). Calcium Binding Affects in Vitro Gastrointestinal Digestion of Bovine α-Lactalbumin under Infant, Adult and Elderly Conditions. Int. Dairy J..

[101] Wang Y., Shi J., Xu Y.-J., Tan C.-P., Liu Y. (2024). The Digestion Fates of Lipids with Different Unsaturated Levels in People with Different Age Groups. Food Chem..

[102] Arnal M., Salcedo L., Talens P., Ribes S. (2024). Role of Food Texture, Oral Processing Responses, Bolus Properties, and Digestive Conditions on the Nutrient Bioaccessibility of Al Dente and Soft-Cooked Red Lentil Pasta. Foods.

[103] Sánchez-García J., Muñoz-Pina S., García-Hernández J., Tárrega A., Heredia A., Andrés A. (2023). In Vitro Digestion Assessment (Standard vs. Older Adult Model) on Antioxidant Properties and Mineral Bioaccessibility of Fermented Dried Lentils and Quinoa. Molecules.

[104] Tsukiashi M., Koyama T., Iwamoto H., Sonoki H., Miyaji K. (2024). Evaluation of the Effect of Thickeners in Enteral Formulas on the Gastric Emptying Rate of Proteins and Carbohydrates Using a Semi-Dynamic Gastric Model. Nutrients.

[105] Chen J., Wu K., Guo W., Guo J., Wang J., Yang X. (2024). Digestion Behavior of Plant-Based Meat Analogs with Anisotropic Fibrous Structure in a Semi-Dynamic Gastric Digestion System. Food Res. Int..

[106] Zaeim D., Liu W., Han J., Wilde P.J. (2022). Effect of Non-Starch Polysaccharides on the in Vitro Gastric Digestion of Soy-Based Milk Alternatives. Food Hydrocoll..

[107] Shani-Levi C., Levi-Tal S., Lesmes U. (2013). Comparative Performance of Milk Proteins and Their Emulsions under Dynamic in Vitro Adult and Infant Gastric Digestion. Food Hydrocoll..

[108] Dupont D., Mandalari G., Mollé D., Jardin J., Rolet-Répécaud O., Duboz G., Léonil J., Mills C.E.N., Mackie A.R. (2010). Food Processing Increases Casein Resistance to Simulated Infant Digestion. Mol. Nutr. Food Res..

[109] Levi C.S., Lesmes U. (2014). Bi-Compartmental Elderly or Adult Dynamic Digestion Models Applied to Interrogate Protein Digestibility. Food Funct..

[110] Hernández-Olivas E., Muñoz-Pina S., Andrés A., Heredia A. (2020). Impact of Elderly Gastrointestinal Alterations on in Vitro Digestion of Salmon, Sardine, Sea Bass and Hake: Proteolysis, Lipolysis and Bioaccessibility of Calcium and Vitamins. Food Chem..

[111] Calvo-Lerma J., Fornés-Ferrer V., Heredia A., Andrés A. (2019). In Vitro Digestion Models to Assess Lipolysis: The Impact of the Simulated Conditions of Gastric and Intestinal PH, Bile Salts and Digestive Fluids. Food Res. Int..

[112] Chen Y., Callanan M., Giblin L., Tobin J., Brodkorb A. (2022). Comparison of Conventional Heat-Treated and Membrane Filtered Infant Formulas Using an in Vitro Semi-Dynamic Digestion Method. Food Funct..

[113] Jiang H., Zhang T., Pan Y., Yang H., Xu X., Han J., Liu W. (2024). Thermal Stability and in Vitro Biological Fate of Lactoferrin-Polysaccharide Complexes. Food Res. Int..

[114] Lajterer C., Shani Levi C., Lesmes U. (2022). An in Vitro Digestion Model Accounting for Sex Differences in Gastro-Intestinal Functions and Its Application to Study Differential Protein Digestibility. Food Hydrocoll..

[115] Wickham M., Faulks R., Mills C. (2009). In Vitro Digestion Methods for Assessing the Effect of Food Structure on Allergen Breakdown. Mol. Nutr. Food Res..

[116] Vardakou M., Mercuri A., Barker S.A., Craig D.Q., Faulks R.M., Wickham M.S. (2011). Achieving Antral Grinding Forces in Biorelevant in Vitro Models: Comparing the USP Dissolution Apparatus II and the Dynamic Gastric Model with Human in Vivo Data. AAPS PharmSciTech.

[117] Minekus M., Marteau P., Havenaar R., Veld J.H.H.I.T. (1995). A Multicompartmental Dynamic Computer-Controlled Model Simulating the Stomach and Small Intestine. Altern. Lab. Anim..

[118] Kong F., Singh R.P. (2010). A Human Gastric Simulator (HGS) to Study Food Digestion in Human Stomach. J. Food Sci..

[119] Kozu H., Nakata Y., Nakajima M., Neves M.A., Uemura K., Sato S., Kobayashi I., Ichikawa S. (2014). Development of a Human Gastric Digestion Simulator Equipped with Peristalsis Function for the Direct Observation and Analysis of the Food Digestion Process. Food Sci. Technol. Res..

[120] Zhong C., Langrish T. (2020). A Comparison of Different Physical Stomach Models and an Analysis of Shear Stresses and Strains in These System. Food Res. Int..

[121] Mennah-Govela Y.A., Swackhamer C., Bornhorst G.M. (2021). Gastric Secretion Rate and Protein Concentration Impact Intragastric PH and Protein Hydrolysis during Dynamic in Vitro Gastric Digestion. Food Hydrocoll. Health.

[122] Chen L., Xu Y., Fan T., Liao Z., Wu P., Wu X., Chen X.D. (2016). Gastric Emptying and Morphology of a ‘near Real’ in Vitro Human Stomach Model (RD-IV-HSM). J. Food Eng..

[123] Feng J., Greco I., Ménard O., Lee J., Jeantet R., Dupont D., Le Feunteun S. (2024). Dynamic in Vitro Gastric Digestion of Skimmed Milk Using the NERDT, an Advanced Human Biomimetic Digestion System. Food Res. Int..

[124] Swackhamer C., Bedane T., Keppler S., Poltorak A., Cheung K., Awais N., Marra F., Bornhorst G.M. (2023). Development and Analysis of a Multi-Module Peristaltic Simulator for Gastrointestinal Research. Food Res. Int..

[125] Peng Z., Wu P., Wang J., Dupont D., Menard O., Jeantet R., Chen X.D. (2021). Achieving Realistic Gastric Emptying Curve in an Advanced Dynamic in Vitro Human Digestion System: Experiences with Cheese—A Difficult to Empty Material. Food Funct..

[126] Madalena D.A., Araújo J.F., Ramos Ó.L., Vicente A.A., Pinheiro A.C. (2023). Assessing the In Vitro Digestion of Lactoferrin-Curcumin Nanoparticles Using the Realistic Gastric Model. Nanomaterials.

[127] Liu W., Zhang T., Jin Y., Li Z., Han J., Tian S. (2024). Development Details of an Artificial Gastric Digestive System (AGDS) and Analysis of Model Food Disintegration and Degradation. Int. J. Food Sci. Technol..

[128] Barros L., Retamal C., Torres H., Zúñiga R.N., Troncoso E. (2016). Development of an in Vitro Mechanical Gastric System (IMGS) with Realistic Peristalsis to Assess Lipid Digestibility. Food Res. Int..

[129] Dang Y., Liu Y., Hashem R., Bhattacharya D., Allen J., Stommel M., Cheng L.K., Xu W. (2020). SoGut: A Soft Robotic Gastric Simulator. Soft Robot..

[130] Payal A., Elumalai A., Murugan S.V., Moses J.A., Anandharamakrishnan C. (2021). An Investigation on Gastric Emptying Behavior of Apple in the Dynamic Digestion Model ARK^®^ and Its Validation Using MRI of Human Subjects—A Pilot Study. Biochem. Eng. J..

[131] Wakita Y., Takahashi M., Tamiya S., Kobayashi I. (2024). Effect of Marination in Lemon Juice on Beef Tenderization and Gastric Digestibility. J. Sci. Food Agric..

[132] Marciani L., Gowland P.A., Fillery-Travis A., Manoj P., Wright J., Smith A., Young P., Moore R., Spiller R.C. (2001). Assessment of Antral Grinding of a Model Solid Meal with Echo-Planar Imaging. Am. J. Physiol. Gastrointest. Liver Physiol..

[133] Wang Z., Kozu H., Uemura K., Kobayashi I., Ichikawa S. (2021). Effect of Hydrogel Particle Mechanical Properties on Their Disintegration Behavior Using a Gastric Digestion Simulator. Food Hydrocoll..

[134] Wang J., Wu P., Liu M., Liao Z., Wang Y., Dong Z., Chen X.D. (2019). An Advanced near Real Dynamic in Vitro Human Stomach System to Study Gastric Digestion and Emptying of Beef Stew and Cooked Rice. Food Funct..

[135] Guo Q., Ye A., Lad M., Dalgleish D., Singh H. (2014). Behaviour of Whey Protein Emulsion Gel during Oral and Gastric Digestion: Effect of Droplet Size. Soft Matter.

[136] Guo Q., Ye A., Lad M., Ferrua M., Dalgleish D., Singh H. (2015). Disintegration Kinetics of Food Gels during Gastric Digestion and Its Role on Gastric Emptying: An in Vitro Analysis. Food Funct..

[137] Guo Q., Ye A., Lad M., Dalgleish D., Singh H. (2016). Impact of Colloidal Structure of Gastric Digesta on In-Vitro Intestinal Digestion of Whey Protein Emulsion Gels. Food Hydrocoll..

[138] Guo Q., Ye A., Lad M., Dalgleish D., Singh H. (2013). The Breakdown Properties of Heat-Set Whey Protein Emulsion Gels in the Human Mouth. Food Hydrocoll..

[139] Wang Z., Kozu H., Uemura K., Kobayashi I., Ichikawa S. (2024). Effect of Mechanical Properties on in Vitro Dynamic Digestion of Starch Contained in Hydrogels. J. Sci. Food Agric..

[140] Mella C., Quilaqueo M., Zúñiga R.N., Troncoso E. (2021). Impact of the Simulated Gastric Digestion Methodology on the In Vitro Intestinal Proteolysis and Lipolysis of Emulsion Gels. Foods.

[141] Lou M., Ritzoulis C., Liu J., Zhang X., Han J., Liu W. (2022). In Vitro Digestion of Tofu with Different Textures Using an Artificial Gastric Digestive System. Food Res. Int..

[142] Shibasaki M., Maeda T., Tanaka T., Sugiyama K., Kozu H., Noguchi R., Umeda T., Araki T., Kobayashi I. (2024). Observation and Analysis of In Vitro Digestibility of Different Breads Using a Human Gastric Digestion Simulator. Foods.

[143] Wang Z., Ichikawa S., Kozu H., Neves M.A., Nakajima M., Uemura K., Kobayashi I. (2015). Direct Observation and Evaluation of Cooked White and Brown Rice Digestion by Gastric Digestion Simulator Provided with Peristaltic Function. Food Res. Int..

[144] Li S., Mungure T., Ye A., Loveday S.M., Ellis A., Weeks M., Singh H. (2024). Intragastric Restructuring Dictates the Digestive Kinetics of Heat-Set Milk Protein Gels of Contrasting Textures. Food Res. Int..

[145] Somaratne G., Ye A., Nau F., Ferrua M.J., Dupont D., Paul Singh R., Singh J. (2020). Egg White Gel Structure Determines Biochemical Digestion with Consequences on Softening and Mechanical Disintegration during in Vitro Gastric Digestion. Food Res. Int..

